# Thymoquinone inhibits cancer metastasis by downregulating TWIST1 expression to reduce epithelial to mesenchymal transition

**DOI:** 10.18632/oncotarget.3973

**Published:** 2015-05-19

**Authors:** Md. Asaduzzaman Khan, Mousumi Tania, Chunli Wei, Zhiqiang Mei, Shelly Fu, Jingliang Cheng, Jianming Xu, Junjiang Fu

**Affiliations:** ^1^ Key Laboratory of Epigenetics and Oncology, the Research Center for Preclinical Medicine, Sichuan Medical University, Luzhou, Sichuan, China; ^2^ Michael E. DeBakey High School for Health Professions, Houston, TX, USA; ^3^ College of Basic Medical Sciences and Institute for Cancer Medicine, Sichuan Medical University, Luzhou, Sichuan, China; ^4^ Department of Molecular and Cellular Biology, Baylor College of Medicine, One Baylor Plaza, Houston, TX, USA

**Keywords:** thymoquinone, cancer metastasis, TWIST1, epithelial to mesenchymal transition, DNA methylation

## Abstract

Proteins that promote epithelial to mesenchymal transition (EMT) are associated with cancer metastasis. Inhibition of EMT regulators may be a promising approach in cancer therapy. In this study, Thymoquinone (TQ) was used to treat cancer cell lines to investigate its effects on EMT-regulatory proteins and cancer metastasis. We show that TQ inhibited cancer cell growth, migration and invasion in a dose-dependent manner. At the molecular level, TQ treatment decreased the transcriptional activity of the *TWIST1* promoter and the mRNA expression of TWIST1, an EMT-promoting transcription factor. Accordingly, TQ treatment also decreased the expression of TWIST1-upregulated genes such as N-Cadherin and increased the expression of TWIST1-repressed genes such as E-Cadherin, resulting in a reduction of cell migration and invasion. TQ treatment also inhibited the growth and metastasis of cancer cell-derived xenograft tumors in mice but partially attenuated the migration and invasion in TWIST1-overexpressed cell lines. Furthermore, we found that TQ treatment enhanced the promoter DNA methylation of the *TWIST1* gene in BT 549 cells. Together, these results demonstrate that TQ treatment inhibits *TWIST1* promoter activity and decreases its expression, leading to the inhibition of cancer cell migration, invasion and metastasis. These findings suggest TQ as a potential small molecular inhibitor of cancer growth and metastasis.

## INTRODUCTION

Cancer metastasis is the major characteristic of malignant carcinomas and is common in late stage of cancer. Cancer is one of the major threats of human health in modern age, and metastasis is thought to be the principal cause of death in cancer patients [1, 2]. Despite the great advances of modern medical science in the last century, metastatic tumors are not yet curable. This is partly because of the complex mechanism of metastasis, and the difficulties in developing efficient treatments. A number of physiological and biochemical pathways is involved in the process of metastasis, detailed mechanisms of which is still far away from clear understanding. Epithelial to mesenchymal transitions (EMT) is a biological process, by which polarized epithelial cells lose their adherence and tight cell–cell junction, enhance migratory capacity, elevate resistance to apoptosis, increase the production of extracellular matrix (ECM) components and gain invasive properties to become mesenchymal cells [1, 3]. Although EMT is a developmental process, it has been utilized in the early stages of cancer to promote invasive metastasis [3]. EMT allows the cancer cells to acquire invasive properties and to develop migratory and invasive characteristics. These events are facilitated by the down-regulation of cell-cell adhesion molecule E-Cadherin, up-regulation of more plastic mesenchymal proteins such as N-Cadherin, Vimentin and smooth muscle actin, deregulation of the Wnt pathway and break through the basement membrane. The transcription factor TWIST1, along with SNAIL1, SLUG and ZEB1, plays the major role in EMT-associated metastasis in cancer cells [3–6]. In addition to the overexpression, epigenetic changes, especially promoter methylation of *TWIST1* gene, are also involved in cancer metastasis [5, 7]. Controlling the expression of TWIST1 and other EMT mediator proteins might be an interesting and potential target in cancer therapeutics.

In traditional or alternative medicinal practices, drugs from natural sources have been used for thousands of years, such as Traditional Chinese Medicine. These natural products are increasingly integrated into modern medical science. The seeds of *Nigella sativa* (black cumin) have a notable place in traditional medicines mainly in Arabian, South Asian, South-East Asian, Mediterranean and some African countries. Ancient Egypt, Turkey, and Greece often used *N. sativa* seeds to treat a myriad of diseases and ailments. *N. sativa* seeds are often named ‘Prophetic medicine’, indicated by the Muslim prophet as a highly potential medicine. Different extracts of *N. sativa* or oils of its seeds possess medicinal properties, and are well known for their anticancer, antihypertensive, antidiabetic, immunomodulatory, analgesic, antimicrobial, anti-inflammatory, spasmolytic, bronchodilative, hepato-protective, renal-protective, gastro-protective and antioxidant activities [8–10]. Biochemical studies have revealed that the major active ingredient behind the medicinal properties of *N. sativa* is Thymoquinone (TQ) [9].

The anticancer activity of TQ is documented, and potential of TQ in controlling cancer metastasis is reported by a few studies [9, 11–13], but the mechanisms behind the antimetastatic role of TQ is still obscure or unknown. This study investigated the role of TQ on the expression of EMT mediator proteins like Cadherins and Vimentin, and their regulatory transcription factors TWIST1, SNAIL1, SLUG and ZEB1 in metastatic cancer cell lines, as well as tumor model of mice. The major objective of our study was to clarify the anticancer and antimetastatic mechanism of TQ in the axis of EMT-cancer cross-linking.

## RESULTS

### Thymoquinone inhibits cancer cell growth, migration, and invasion

**Figure 1:**
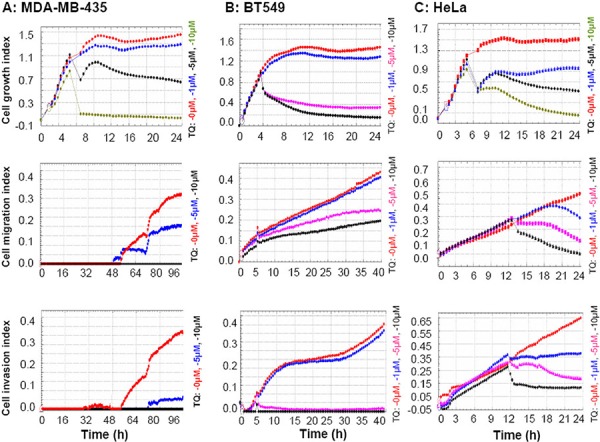
Thymoquinone (TQ) inhibitions on cancer cell growth, migration and invasion Cells were analyzed by real time cell analyzer after treating with different concentrations of TQ. TQ dose dependently inhibited the growth, migration and invasive characteristics of MDA-MB-435 **A.** BT549 **B.** and HeLa **C.** cells.

To investigate the TQ effects on cancer cell growth, migration and invasion, the cell index results were evaluated by real time cell analyzer, which showed that TQ inhibited the growth, migration and invasive characteristics in all of the three tested cancer cell lines MDA-MB-435, BT549 and HeLa. TQ was found effective even at a low concentration (1 μM), and the inhibition was found highly significant at a concentration of 5 μM (Figure 1). MTT analysis confirmed the dose dependent cytotoxic activity of TQ against these cancer cell lines (Supplementary Figure S1).

### Thymoquinone regulates the expression of EMT associated proteins

**Figure 2:**
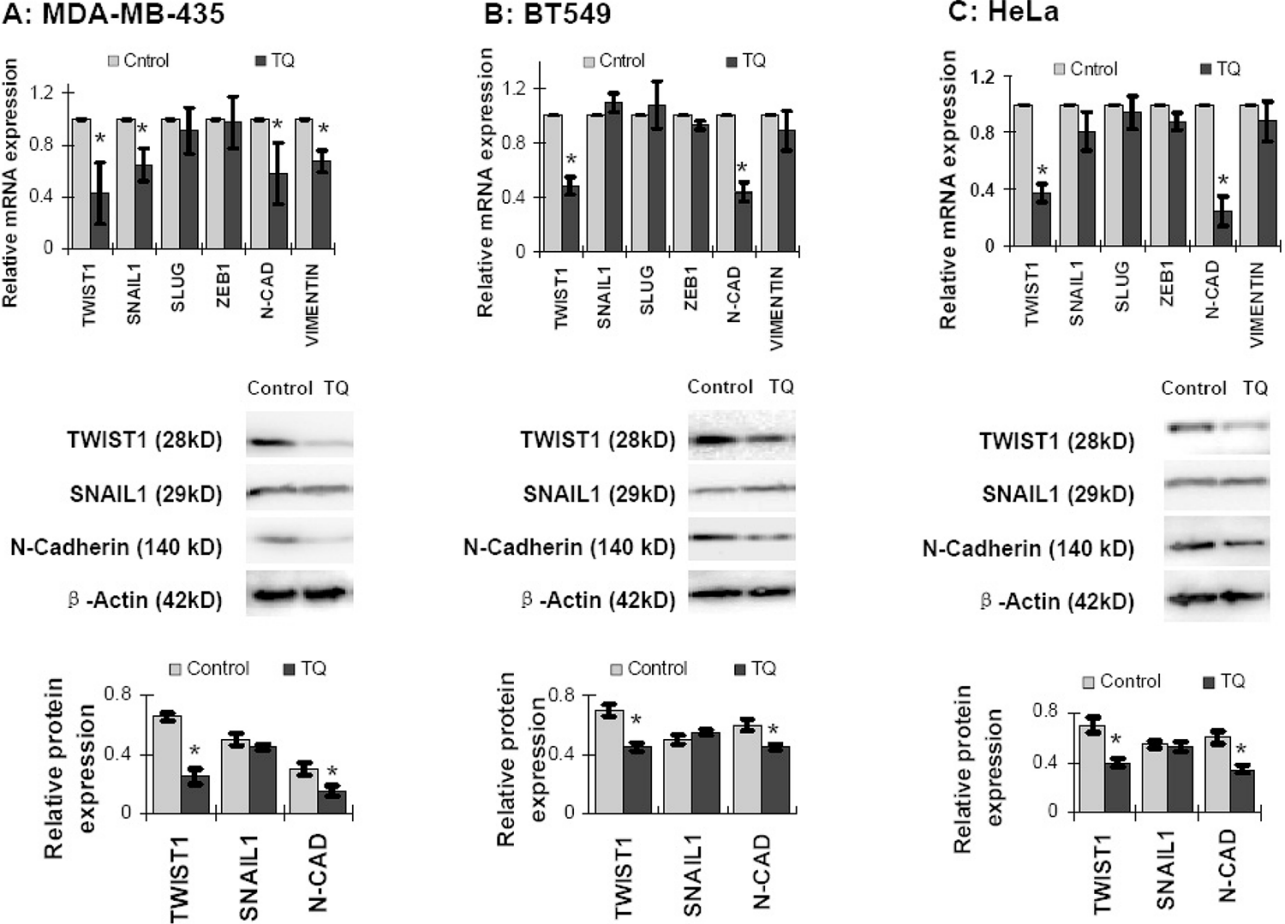
Effects of Thymoquinone (TQ) on mRNA and protein level expression of EMT associated proteins in cancer cells TQ treatment (5 μM) for 6 h inhibited the expression of TWIST1 and N-Cadherin in MDA-MB-435 (**A.** upper panel), BT549 (**B.** upper panel) and HeLa (**C.** upper panel) cells. Some extents of inhibition were also observed in SNAIL1 and Vimentin expression, especially in MDA-MB-435 cells (A: upper panel). But, TQ showed no significant change in ZEB1 and SLUG expression. Again, TQ treatment (5 μM) for 48 h inhibited the protein level expression of TWIST1 and N-Cadherin MDA-MB-435 (A: middle and lower panel), BT549 (B: middle and lower panel) and HeLa (C: middle and lower panel) cells. However, SNAIL1 expression was found changed non-significantly. Results are expressed as mean ±SD (*N* = 3); *P < 0.05.

In order to investigate the TQ effect on the expression of EMT associated proteins, cancer cells were treated with 5 μM of TQ for 6 hours, and then total RNA was extracted from cells for quantitative real time PCR (qPCR). The qPCR analysis showed that TQ significantly inhibited the mRNA level expression of TWIST1 and N-Cadherin in all of the MDA-MB-435, BT549 and HeLa cell lines (*P* < 0.05) (Figure 2). Some extents of reduction in SNAIL1 and Vimentin were also observed, especially in MDA-MB-435 cells by TQ treatment; however, no significant change in SLUG or ZEB1 expression was observed (Figure 2). To determine protein expression, cancer cells were treated with 5 μM of TQ for 48 hours, and proteins were extracted for Western blot analysis. Like mRNA level expression, TWIST1 and N-Cadherin expressions at protein level were inhibited by TQ treatment in MDA-MB-435, BT549 and HeLa cell lines; however, no significant change in SNAIL1 was observed (*P* > 0.05) (Figure 2). Both of the mRNA and protein levels of E-Cadherin were expressed minimally or completely unexpressed in these three cancer cell lines. We speculated that TQ treatment decreases the expression of TWIST1 and its downstream protein N-Cadherin in cancer cells, while SNAIL1 and Vimentin were partially affected in cell specific manner.

### Regulation of migration and invasion by Thymoquinone in TWIST1-knockdown and TWIST1-overexpressed inducible cells

**Figure 3:**
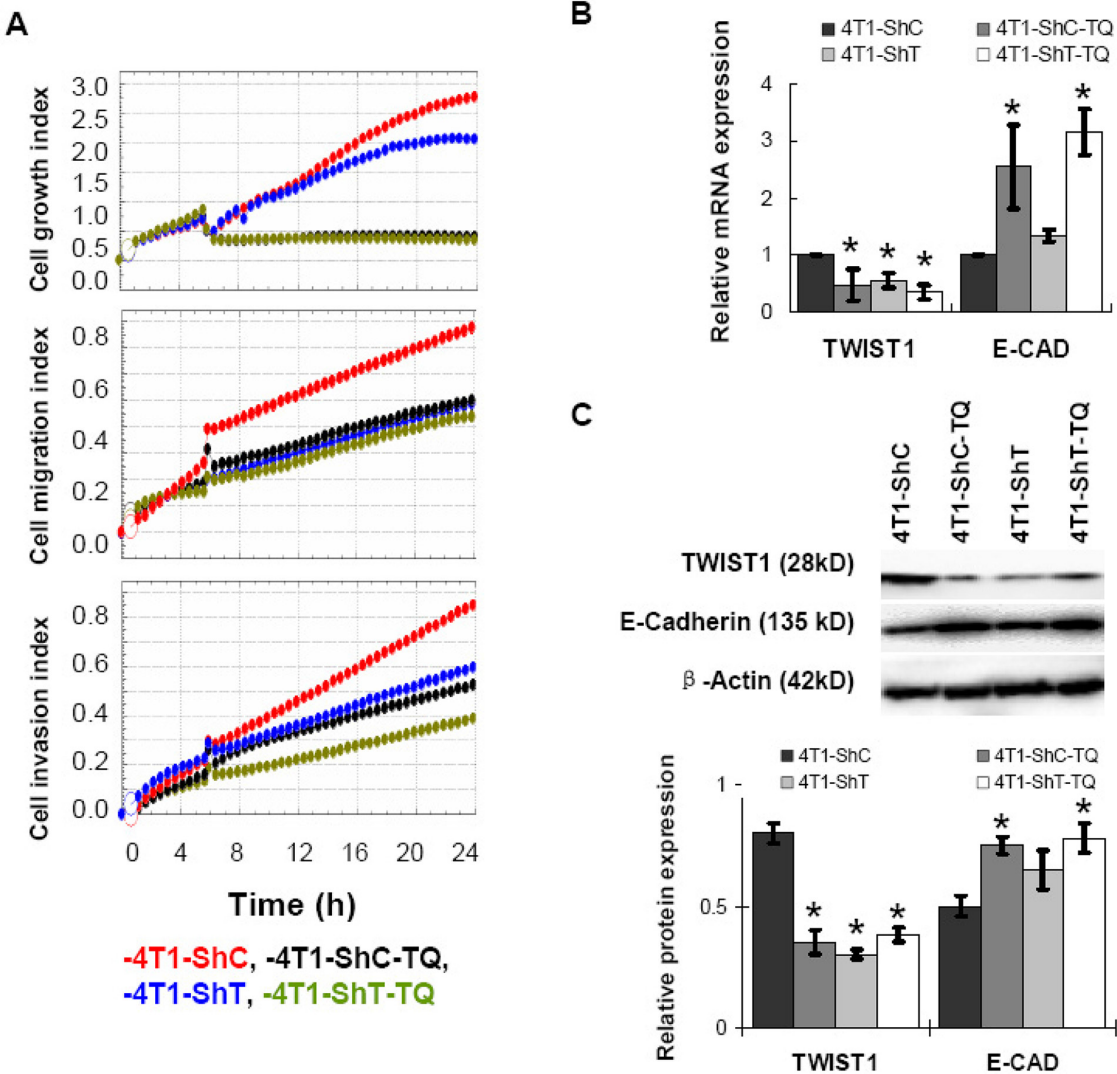
Effects of Thymoquinone (TQ) on the TWIST1-knockdown 4T1 cancer cells The cellular growth rate, migration and invasion were reduced in knockdown (4T1-ShT) cells than control (4T1-ShC) cells **A.** TQ treatment also reduced the 4T1 cell growth, migration, and invasion (A). After 6 h of TQ treatment (5 μM), the mRNA level, and after 48 h of treatment, the protein level expressions of TWIST1 were reduced significantly in both of the control and knockdown cells **B, C.** With the decrease of TWIST1, E-cadherin expression was increased by TQ treatment (B, C). Results are expressed as mean ±SD (*N* = 3); *P < 0.05.

TWIST1 has been regarded as an oncogene [14], and knockdown of TWIST1 is expected to be beneficial against cancer. In this study, *TWIST1* gene was knocked down in 4T1 mouse breast cancer cells by shRNA technology. Then, TWIST1-knockdown cells (4T1-ShT) and control (4T1-ShC) cells were treated with TQ (5 μM) for the investigation of cell growth, migration, and invasion index by real time cell analyzer. Results showed that the cellular growth, migration, and invasion rates were reduced in knockdown (4T1-ShT) cells than in control (4T1-ShC) cells (Figure 3). TQ treatment also reduced and even potentiated TWIST1 knockdown leading to an inhibition of 4T1 cell growth, migration and invasion (these characteristics were found lower in 4T1-ShC-TQ and 4T1-ShT-TQ cells) (Figure 3). For the investigation of the expression of TWIST1 and its downstream proteins, after 6 h of TQ treatment (5 μM), the mRNAs and after 48 h of TQ treatment, the proteins were extracted from cancer cell lines. The qPCR and Western blot analysis showed that TWIST1 expression was reduced more significantly in both of the control and knockdown cells (4T1-ShC-TQ and 4T1-ShT-TQ) than in 4T1-ShC and 4T1-ShT cells respectively (*P* < 0.05). Interestingly, E-Cadherin expression was increased in TQ-treated cells (in an opposite manner to TWIST1 expression). However, TWIST1 knockdown showed an increase of the E-Cadherin expression less than the TQ-treated cells (Figure 3). Both of the mRNA and protein levels of N-Cadherin were expressed minimally or completely unexpressed in 4T1 cell lines (data not shown). Thus, we report that TQ consistently reduced the expression of TWIST1, which is associated with cancer cell migration and invasion.

**Figure 4:**
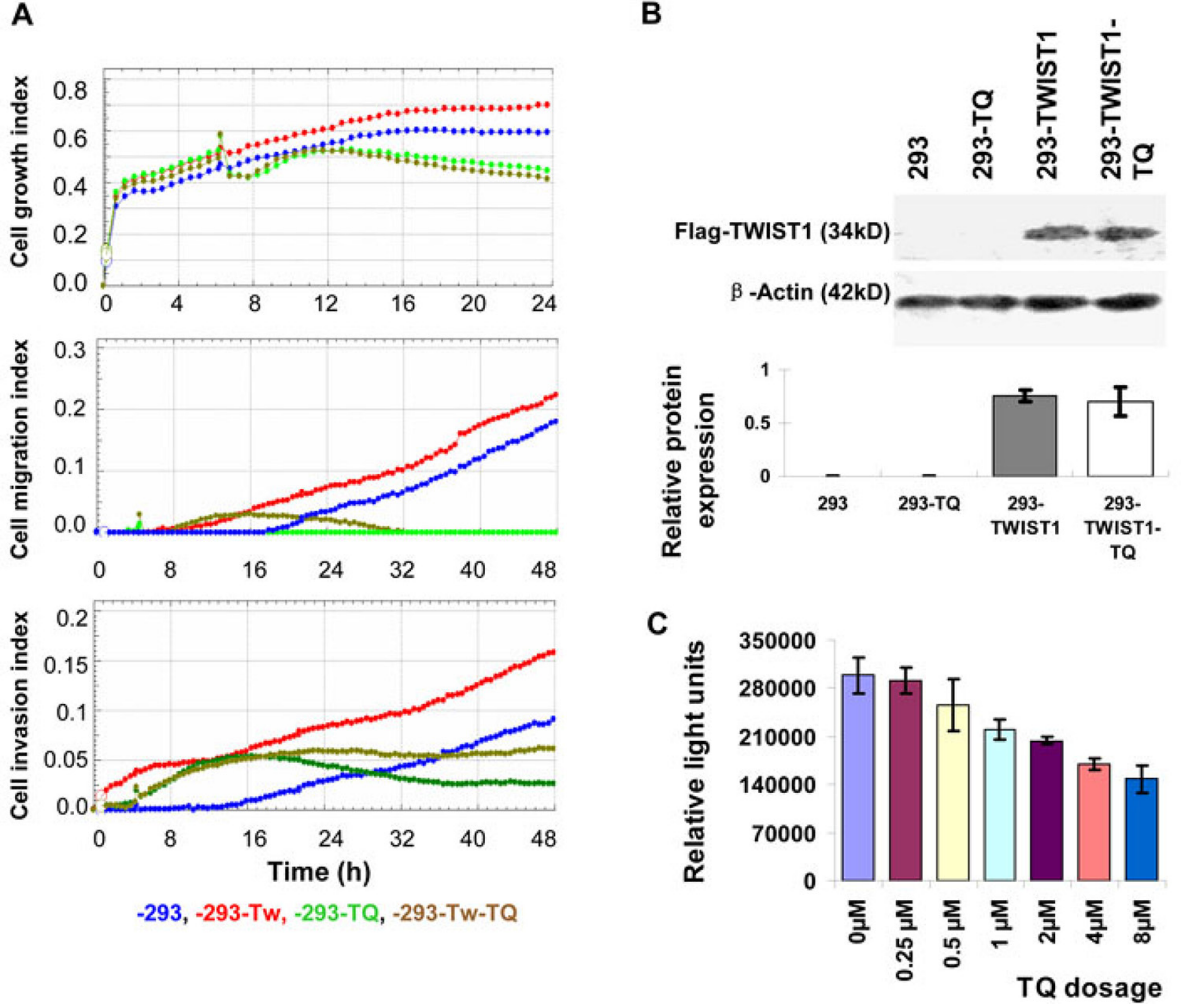
Effects of Thymoquinone (TQ) on the TWIST1-overexpressed cells The index of growth, migration, and invasion in *TWIST1* over-expressed inducible HEK293 cells (−293-TWIST1, −293-TWIST1-TQ) and control cells (−293, −293-TQ) with DOX induction **A.** TWIST1 expression in inducible HEK293 cells was not affected by TQ treatment **B.** Luciferase reporter assay showed that TQ decreased the TWIST1 reporter activity in Hela cells, as the Relative Light Units (RLU) was decreased with the increase of TQ dosage **C.**

Endogenous TWIST1 expression in HEK293 cells is very low or undetectable. TWIST1 was experimentally overexpressed in inducible HEK293 cell lines, which have been reported in our previous study [15]. This increased TWIST1 level showed some extent of migratory and invasive characteristics, although normal HEK293 had very little migratory and invasive properties (also indicated in a previous study [16]). There was no significant change observed in cell growth pattern when TWIST1 was overexpressed (Figure 4A). Interestingly, TQ treatment at the first ∼10 hours (from the time ∼6 to ∼16 h) did not block cellular migration and invasion in HEK293-TWIST1 cells (Figure 4A, yellow lines), but TQ treatment reduced the abilities of cellular migration and invasion later (Figure 4A). Meanwhile, TQ had little or no effect on ectopic TWIST1 expression (Figure 4B). This indicates that TQ treatment in TWIST1-overexpressed inducible HEK293 (293-TWIST1) cells attenuated the cell migration and invasion partially, without inhibition of TWIST1 expression.

### Thymoquinone directly targets *TWIST1* gene

To investigate whether TQ directly targets *TWIST1* gene, luciferase reporter assay was performed. The TWIST1 reporter gene was transfected with or without TQ treatment into HeLa cells, and after 48 h of transfection, luciferase activity was measured and the results showed that TQ dose dependently decreases the luciferase activity (Relative Light Units, RLU) of TWIST1 promoter reporter gene (Figure 4C). This indicates that TWIST1 might be a direct target of TQ.

### Effects of Thymoquinone on cancer cell-derived xenograft tumors in mice

**Figure 5:**
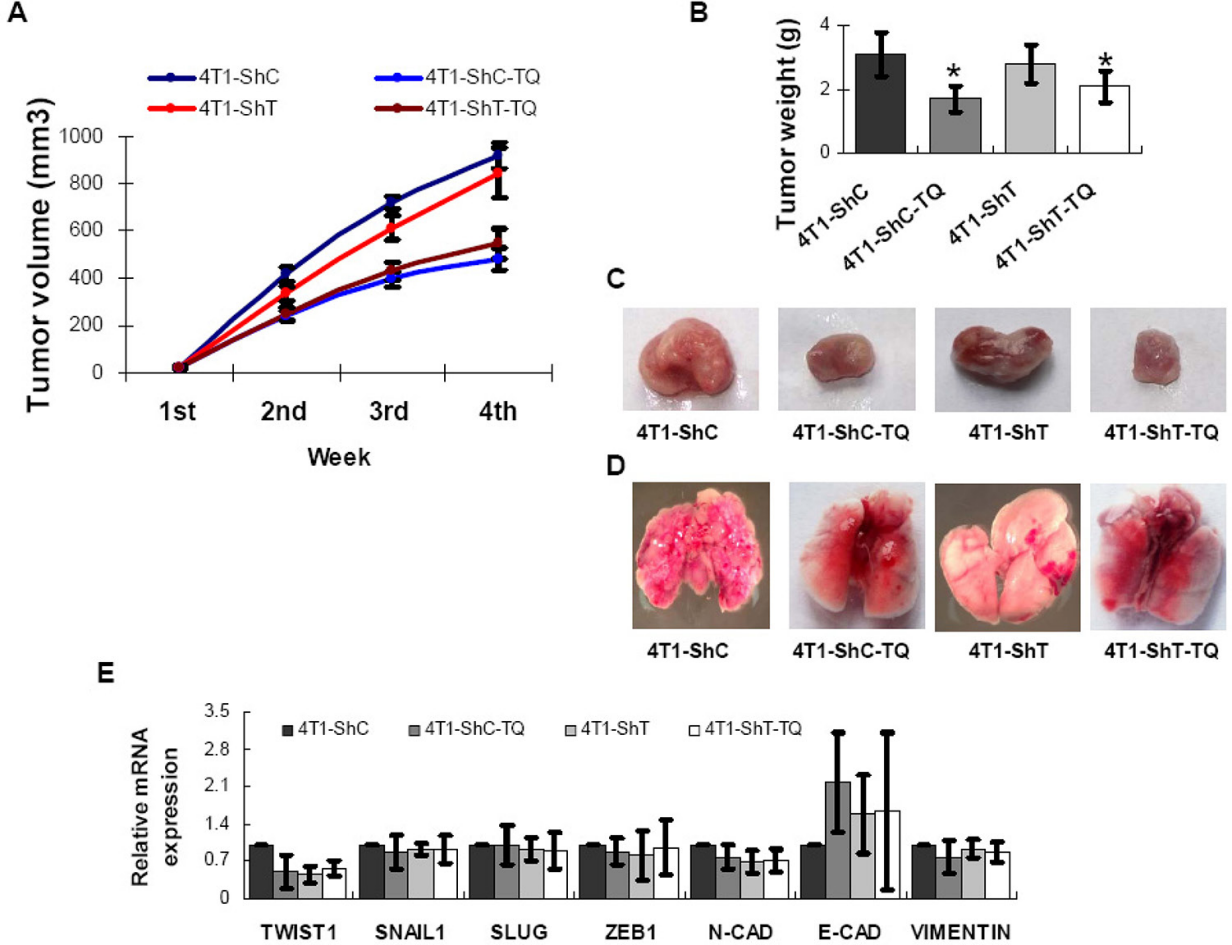
Inhibitions of Thymoquinone (TQ) on breast cancer model of mouse TQ treatment inhibited the cancer growth in mice, while *TWIST1* knockdown inhibited cancer growth by only some extents **A, B, C.** However, *TWIST1* knockdown reduced the cancer metastasis, as tumors metastasized to lung in 4T1-ShC group of mouse, but not in 4T1-ShT cells **D.** TQ treatment also inhibited cancer metastasis to lung (D). The mRNA level expression of TWIST1 and N-Cadherin were found to be decreased and E-Cadherin was found to be increased by TQ treatment in mouse breast cancer tissue **E.** Results are expressed as mean ±SD (*N* = 5); *P < 0.05.

Four groups of mice were injected with 4T1 cells (two with 4T1-ShC and two with 4T1-ShT) and fed (with or without TQ) for 4 weeks, and then sacrificed. 10 mg/kg body weight/day was used as TQ dosage, because previous studies indicated that the LD_50_ of TQ was 105 mg/kg and 870.9 mg/kg after intraperitoneal injection and oral gavages respectively and TQ was effective as anticancer molecule at even 4 mg/kg body weight/day [17]. Tumor growth pattern in mice showed that TQ treatment for 3 weeks efficiently inhibited the tumor growth in female BALB/c mice (4T1-ShC-TQ and 4T1-ShT-TQ groups), but TWIST1 knockdown inhibited tumor growth by only some extents (Figure 5). Tumor weight was also found significantly lower in 4T1-ShC-TQ and 4T1-ShT-TQ groups (*P* < 0.05). Cancer was metastasized into lung in 4T1-ShC group, which was reduced in 4T1-ShT group. This indicates that TWIST1 knockdown reduced metastasis in breast cancer model of mouse. Interestingly, TQ treatment also inhibited cancer metastasis to the lungs (in 4T1-ShC-TQ and 4T1-ShT-TQ groups) (Figure 5). Thus, our findings report the anticancer and antimetastatic activity of TQ both *in vitro* and *in vivo*. The mRNAs were extracted from mouse cancer tissues for the analysis of the expression level by qPCR. TWIST1 and N-Cadherin were found down-regulated and E-Cadherin was found up-regulated by TQ treatment; however, the data was found statistically insignificant (*P* > 0.05) (Figure 5). This might be due to the highly variable physiological systems in individual mice.

### Effects of Thymoquinone on *TWIST1* promoter methylation in cancer cells

**Figure 6:**
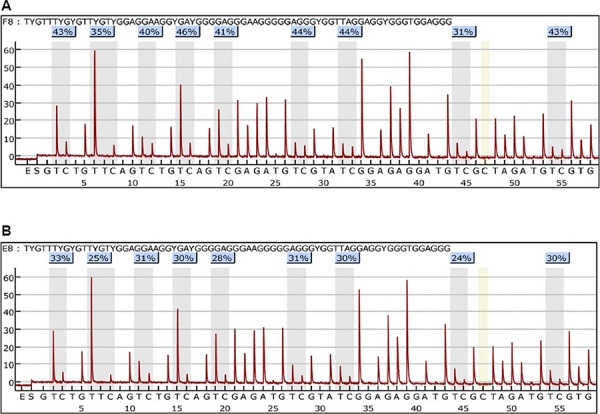
Effects of Thymoquinone (TQ) on methylation of the proximal promoter in *TWIST1* gene in BT549 cells Treatment of TQ (5 μM) for 24 h increased the methylation level of *TWIST1* proximal promoter in TQ treated cells **A.** more than in non-treated cells **B.** The number of the percentage indicated the DNA methylation level on the CpG islands.

By the pyro-sequencing, the proximal promoter methylation of *TWIST1* gene on the CpG islands was found increased by TQ treatment (5 μM for 24 h) in BT549 cells (Figure 6), however, little or no change in HeLa and MDA-MB-435 cells was observed (Supplementary Figure S2). Thus, promoter methylation of *TWIST1* gene might be one of the mechanisms of TWIST1 down-regulation by TQ, which might be a cancer-cell-specific mechanism. To further investigate the change of the expressions of DNA methylating enzymes-DNA methyl transferases (DNMT1, DNMT3a), qPCRs were performed, but the results showed that the expressions of DNMT1, DNMT3a were not significantly changed (*P* > 0.05) (Supplementary Figure S3). Western blot analysis was also performed for the determination of DNMT1 protein in BT549 cells treated with 5 μM of TQ or without TQ, which further confirmed the qPCR results (Supplementary Figure S3).

## DISCUSSION

Metastasis is a process through which cancer cells spread from the place where it originates to other places in the body. By this process, cancer becomes more dangerous and potentially lethal. Although the number of cancer survivors continues to increase due to the longevity and growth of the population and improvements in early detection and early treatment [18], metastatic cancers are among the major health burdens, and one of the main causes of death currently. Controlling metastasis is a major issue in cancer therapeutics now-a-days. Cancer cell migration and invasion are the two major characteristics in the process of metastasis. In this study, we have found that TQ dose dependently inhibited the cancer cell migration and invasion as well as cellular growth. Moreover, in breast tumor model of mouse, TQ inhibited cancer growth and lung metastasis. The anticancer and antimetastatic activities of TQ have been previously reported by some studies [11–13, 19–24], however, the mechanisms of antimetastatic role of TQ is extremely complex and still obscure.

We investigated the role of TQ on EMT mediators in cancer cells, as several studies indicated that aberrant expression of EMT mediators are associated with cancer metastasis. Our study revealed that TQ down-regulates TWIST1 and its target protein N-Cadherin (the cell adhesion molecule) in MDA-MB-435, BT549 and HeLa cell lines. Interestingly, both of TWIST1 and N-Cadherin are usually high expressed in metastatic cancer cells [5, 25–27]. TWIST1 has been indicated as oncoprotein [14], which, with the support of other transcription factors, like SNAIL1, SLUG and ZEB1, regulates the expression of Cadherins and some other proteins involved in both of EMT and metastasis process, as well as cancer initiation and progression [5, 6, 15, 16, 28–32]. Another protein, E-Cadherin is usually suppressed in cancer cells [3, 5, 6, 26, 27, 33] but has been up-regulated in 4T1 mouse breast cancer cell line by TQ treatment. The results of this study thus showed that TQ down-regulates TWIST1 in cancer cells, and also regulates the Cadherins (up-regulation of E-Cadherin and down-regulation of N-Cadherin), concomitantly with its antimetastatic activity. Generally, over-expression of N-Cadherin and low-expression of E-Cadherin are the hallmarks of EMT and metastasis, and TWIST1 plays a key role behind this. Our study revealed that TQ can reverse this Cadherin balance by down-regulating TWIST1 in cancer cells. *In vivo* study also indicated the tumor growth and metastasis inhibition by TQ treatment in cancer cell-derived xenograft tumors in mice. These findings clearly indicate that TQ is a potential anticancer and antimetastatic compound, which might act via targeting the EMT proteins, especially TWIST1.

Some studies have indicated that knockdown of TWIST1 in cancer cell lines inhibits cancer initiation progression and metastasis [4, 15, 34]. We have used the TWIST1-knockdown 4T1 cell line for TQ treatment. Both *in vitro* and *in vivo* studies of our investigation revealed that knockdown of TWIST1 are associated with the inhibition of migration and invasion of cancer cells. Treatment of TQ in TWIST1 knockdown cancer cells induced inhibition activity of migration and invasion. TWIST1 knockdown also made little changes in E-Cadherin expression, but TQ up-regulated E-Cadherin significantly. This indicates that TQ may up-regulate E-Cadherin by some other mechanisms, in addition to modifying TWIST1.

A previous study reported that TQ targets Akt pathway, while promoting antimetastatic activity [11]. Another study indicated Wnt pathway as the target of TQ [35]. Interestingly, Akt pathway or Wnt pathway are directly related to the TWIST1 pathway in the progression of cancer metastasis [36–38]. In our study, we also have checked the Akt and Wnt expression, and found that these are modified by TQ treatment in cancer cells (Supplementary Figure S4).

TWIST1 was overexpressed in inducible HEK293 cell lines due to very low or undetectable endogenous TWIST1 expression in HEK293 cells [15]. The increased TWIST1 level showed some extent of migratory and invasive characteristics, although normal HEK293 had very little migratory and invasive properties [15, 16]. Interestingly, TQ treatment did not block cellular migration and invasion in HEK293-TWIST1 cells at the first ∼10 h (from the time ∼6 to ∼16 h) (Figure 4A, yellow lines); later, TQ treatment blocked cellular migration completely and invasion partially or some extents (Figure 4). However, TQ showed no significant inhibition on the ectopic TWIST1 expression (Figure 4B). This indicates that TQ treatment in 293-TWIST1 cells attenuated the cell migration and invasion partially, which supports the connection: TQ-TWIST1 in the axis of EMT-cancer cross-linking. However, after 10 h TQ treatment, the abilities of cellular migration and invasion in HEK293-TWIST1 cells were reduced. This indicates that TQ effects on cellular migration and invasion are dependent on TWIST expression, but later does the same to other pathways too, such as Akt pathway or Wnt pathway, which we also found was modified by TQ treatment in cancer cells (Supplementary Figure S4). Meanwhile, the lucifease assay confirmed that TQ can directly interfered with *TWIST1* promoter reporter gene activity. Therefore, our study indicates that TWIST1 might be a direct target of TQ; however, other molecules/pathways might also be involved later. As metastasis is a complex biochemical and physiological process with several molecular pathways involved in this process, we speculate that TQ targets one or more signaling molecules in these pathways, and TWIST1 might be an important target by TQ for its role of anticancer and antimetastasis.

Promoter methylation of *TWIST1* gene has been reported in several cancers, especially in distant metastasis [39–41]. Interestingly, methylation of *TWIST1* promoter is associated with protein expression in tumor stroma to influence the epithelial-mesenchymal transition-like tumor budding phenotype in colorectal cancer [40]. However, no clear association was found between *TWIST1* promoter methylation and TWIST1 expression in breast cancer [39]. In our study, we have found the promoter region of *TWIST1* gene in MDA-MB-435 and HeLa cancer cell lines are highly hypermethylated, and TQ treatment did not make any change in methylation level of *TWIST1* promoter in these two cell lines. However, TQ treatment increased the methylation level at all of the CpG islands of *TWIST1* promoter in BT549 cells, which we speculate that TQ treatment might be associated with TWIST1 expression. This promoter methylation of *TWIST1* by TQ is most likely cancer cell-specific, and might be associated with *TWIST1* down-regulation. DNA methylating enzyme DNA methyl transferases 1 (DNMT1) is necessary in maintaining global methylation and aberrant CpG island methylation in human cancer cells [42], whereas the *de novo* DNA methyltransferase 3a (DNMT3a) is responsible for the establishment of *de novo* genomic DNA methylation patterns, involving in normal development and in many diseases including cancer [43]. So the qPCR and Western blot analysis were performed to examine the mRNA and protein expression of DNMT1. However, results showed that both of DNMT1 and DNMT3a were not significantly changed by TQ treatment in the tested cancer cell lines. We speculate that DNA methylation status is somehow modified by TQ and TQ might play a role in *TWIST1* promoter methylation in cell specific manner or through unknown pathways. Thus, the epigenetic role of TQ on TWIST1 and other EMT-associated proteins is also interesting for our future research.

In conclusion, we suggest TQ, a potential anticancer and antimetastatic natural compound, might be used for further clinical studies and applications in cancer therapeutics. Our study indicates that TWIST1 along with its down-stream proteins, especially Cadherins are the novel targets of TQ treatment in cancer cells. This study also suggests that the epigenetic modification of *TWIST1* promoter might be associated with TWIST1 down-regulation by TQ treatment.

## MATERIALS AND METHODS

### Ethics statement

Investigation for animal model has been conducted in accordance with the ethical standards and according to the Declaration of Helsinki and according to national and international guidelines. The protocol has been approved by the Sichuan Medical University review board.

### Cell culture and Thymoquinone treatment

The inducible HEK293 cell line with over-expressed TWIST1 (293-TWIST1) was described previously [15]. Human cancer cell lines MDA-MB-435, HeLa and BT549, and mouse breast cancer cell line 4T1 were cultured in DMEM or RPMI1640 media (Thermo Fisher Scientific, USA) with 10% fetal bovine serum (FBS) (Hangzhou Sijiqing Biological Engineering Materials Co., Ltd., China). TQ was purchased from Sigma-Aldrich (St. Louis, MO, USA) and suspended in dimethyl sulfoxide (DMSO). Different concentrations of TQ were used to treat cancer cell lines, while DMSO was used as control.

### Cell growth, migration and invasion assay

A real time cell analyzer (xCELLigence RTCA DP, Roche, Germany) was used for the real time analysis of cell migration, invasion and growth index [15]. 100 μl of cell suspensions (5 × 10^4^ cells/ml) were seeded on each of the 16 well E-plate for cell growth index. CMI plates were used for cell migration and invasion index analysis, where the lower chamber wells were filled with chemotaxis inducer (10% serum supplemented media), and 100 μl of cell suspensions (5 × 10^4^ cells/ml) in serum free medium were added into the wells of upper chamber. For cell invasion assay, the membrane of the CMI plate was pre-coated with Matrigel (354277, BD Biosciences) with 1:40 diluted in 1 × PBS before cells were seeded. After a certain period of cell growth (4–12 h, indicated in the figures), TQ of different concentrations (1–10 μM) were added into the wells. The process of cell migration and invasion was monitored every 30 min till the experimental endpoint. Cell viability was examined by MTT [3-(4, 5-Dimethylthiazol-2-yl)-2, 5-diphenyltetrazolium bromide, a tetrazole] assay. Briefly, in a 96-well cell culture plate, 1 × 10^5^ cells (in 100 μl media) were plated per well with different concentrations of TQ (1–50 μM), and incubated for 24 h. Then 10 μl of MTT reagent was added and incubated for 3 h until purple precipitate was visible. The upper solution (media + MTT) was then removed carefully, and 100 μl of detergent (DMSO) was added into each well. The plate was kept in the dark at room temperature for 2 h, and then absorbance (optical density) was recorded at 570 nm in a microplate spectrophotometer (Multiskan™ GO, Thermo Scientific, USA).

### RNA extraction, RT-PCR and qPCR analysis

After TQ treatment for 6 h, cellular total-RNA was extracted by using RNeasy^®^ mini kit (Cat No: 74104, Qiagen), following the manufacturer's protocol. RNA concentration was measured by using ND-1000 UV/Vis spectrophotometer (NanoDrop, USA) and final concentration was set as 150 ng/μl for cDNA synthesis (reverse transcriptase/RT-PCR). In a 10 μl of RT reaction system, 2 μl of 5 × RT buffer, 1 μl of dNTPs, 0.5 μl of random primer, 0.5 μl of Rev. Ace (enzyme, purchased from TOYOBO and BIOBRK companies of China), 0.25 μl of super RI, 0.25 μl of RT-enhancer, 2.25 μl of RNase free water and 3.25 μl of RNA (150 ng/μl) were taken. The reaction was completed in a thermocyler (Mastercycler gradient, Eppendorf, Germany) with the following steps: 10 min at 30°C, 30 min at 42°C, 5 min at 99°C, 5 min at 4°C, followed by final holding at 16°C. The synthesized cDNAs were then diluted by adding 40 μl ddH_2_O, and used as templates for quantitative PCR (qPCR). The sequence-specific fluorescence-labeled probes and primers for Taqman qPCR were matched by the Universal Probe Library Center (Roche, Germany) [15, 32, 44, 45]. The primer sequences for the investigated RNA of precursor genes are presented in Supplementary (Table 1 & 2). 18S RNA was used as internal control. In a 10 μl of the reaction system, 5 μl of 2 × PCR-probe mix, 0.02 μl of probe, 1 μl of primers, 2 μl of H_2_O and 2 μl of cDNA were mixed, and reaction was completed in StepOne plus Thermocycler (Applied Biosystem) with a 40 cycle of amplification according to the manufacturer's protocol. Relative contents of mRNA were obtained by normalization to 18 S RNA, and were calculated and expressed as 2^−ΔΔCT^.

### Protein extraction and western blot analysis

After TQ treatment for 48 h, cellular proteins were extracted by using EBC lysis buffer [46]. Proteins were then separated on vertical polyacrylamide gel electrophoresis (PAGE), and transferred to nitrocellulose membrane. The membrane was kept in 5% milk (in 1 × TBST) at 4°C for 2 h, and then incubated with primary antibody solution at 4°C for 12 h with gentle shaking. The membrane was then washed thrice with TBST, and incubated with secondary antibody tagged with horseradish peroxidase (HRP) for 4∼8 h at room temperature with gentle shaking. The membrane was again washed thrice with TBST, and protein bands were visualized after the chemiluminiscence reaction by using a digital imaging system (Universal Hood II, Bio-Rad Lab, Italy) [45, 46]. The primary antibodies used in this study were anti-TWIST1 (Abcam), anti-SNAIL1 (Santa Cruz Biotechnology, Inc), anti-N-Cadherin (Cell Signaling Technology), anti-E-Cadherin (Cell Signaling Technology), Rabbit Anti-DNMT1 Polyclonal Antibody (bs-0678R, Bioss Inc) and as control, anti-beta actin (Beyotime Biotechnology). Corresponding to primary antibodies, anti-mouse (Bioworld technology) and anti-rabbit antibodies (Beyotime Biotechnology) were used as secondary antibody. The comparative level of protein expression was measured by analyzing the visualized protein bands using ImageJ software (National Institute of Health, USA).

### Generation of TWIST1-knockdown and TWIST1-overexpressed cell lines

*TWIST1* gene was knocked down in 4T1 cells by using shRNA technology [47]. The lentiviral vector construct pLKO-Tet-On that contains all the necessary cis-elements for packaging, reverse transcription, and integration, were used for the inducible expression of shRNA in target cells. In the absence of doxycycline (DOX), shRNA expression was repressed by constitutively-expressed TetR protein. Upon the addition of DOX to the culture media, shRNA expression was triggered resulting in *TWIST1* gene knock-down [15, 47]. TWIST1 Knockdown cells (4T1-ShT) and control (4T1-ShC) cells were treated with TQ for the investigation of cell growth, migration and invasion index, as well as mRNA and protein expression of TWIST1, N-Cadherin and E-Cadherin. Also, we have constructed an inducible HEK293 cell line with over-expressed TWIST1 (293-TWIST1) [15], and treated with TQ for the investigation of cell growth, migration and invasion index, as well as protein expression of TWIST1. In this case, flag-tagged *TWIST1* gene was transfected into HEK293 cells and inducible expressed by treating with DOX. During Western blot analysis for protein expression determination, flag antibody was used for the measurement of ectopic TWIST1 expression.

### Luciferase reporter assay

Luciferase reporter assay was performed by using *TWIST1* promoter reporter gene which has been reported previously [31, 48]. 40%-confluent HeLa cells in 12-well plates were transfected with 100 ng of the pGL3-hTWIST1-Luc promoter/reporter plasmid in each well without or with indicated different concentration of TQ (0.25–4 μM), and the activity was measured by using Luciferase Assay System (Promega, USA). The relative luciferase activity, expressed as ‘Relative Light Units’ (RLU) was determined by using 3010Luminometer (BD Monolight™, USA) after two days of transfection. All experiments were performed in triplicates.

### Generation of breast tumor model of mouse and *in vivo* study of TQ effects

Twenty female BALB/c mice (5 weeks of old) [15] were divided into 4 groups. Group 1 and group 2 were injected with 4T1-ShC cells (2 × 10^5^ cells in 1 × PBS with a total volume of 100 μl), and group 3 and group 4 were injected with 4T1-ShT cells (2 × 10^5^ cells in 1 × PBS with a total volume of 100 μl). 1% Phenobarbital sodium (in 1 × PBS) was used as anesthetic. Cancer cells were injected into the both sides of the mouse mammary fat pad [15]. Mice were fed with normal diet and drinking water regularly. From the next week, when tumors appeared, group 2 mice were being fed with TQ (10 mg/kg body weight/day), and group 3 mice were being fed with 0.5 mg/mouse/day of DOX with drinking water, while group 4 mice were being fed with both of the DOX and TQ. Mice were fed for another three weeks, and tumor volume was measured by using slide calipers on each week. For measuring tumor volume, the following formula was applied:

$$\text{Volume }(\text{A})=1/2\;[\text{Length}\times {\text{Width}}^{2}]$$

After four weeks of cancer cell injection, mice were sacrificed, and tumor tissues were collected. Lungs of the mice were also collected to observe the metastasis of cancer into lungs. Total RNA materials were extracted from tumor tissues for the analysis of mRNA level expression by qPCR.

### Gene methylation assay

MDA-MB-435, HeLa and BT549 cells were treated with TQ (5 μM) for 24 h, and DNA was extracted by using TIANamp genomic DNA kit (TianGen, China). The PCR products from bisulfite-treated genomic DNA samples were analyzed with Pyrosequencing technology, in order to quantify the site-specific methylation. The Qiagen bisulfite kit was used for the treatment of genomic DNA and the primers used for the amplification of *TWIST1* gene promoter by PCR were as follows: F: GGGAGAGATGAGATATTATTTATTGTGT; R: CTCCTCCCAAACCATTCAA. The sequencing samples were prepared with the Vacuum Prep workstation (Biotage AB, Uppsala, Sweden) according to the following protocol: 40 μl of the amplicons, 3 μl streptavidin Sepharose HP beads (GE Healthcare), 37 μl binding buffer (10 mmol/l Tris-HCl, 2 M NaCl, 1 mmol/l EDTA, 0.1% Tween-20, and 15 μl Milli-Q (18.2 MΩ × cm) water, pH 7.6) were mixed and kept for 5–10 min. The biotinylated amplicons were immobilized onto the streptavidin sepharose beads and then passed through one denaturation step and two washing steps using the Vacuum Prep workstation. The amplicons were transferred to a plate containing 0.4 μmol/l sequencing primer (AGGAGGGGAAGGAAA) in 40 μl annealing buffer (20 mmol/l Tris-acetate, 2 mmol/l magnesium acetate, pH 7.6). Then the plate was heated with the samples at 80°C for 2 min. Pyrosequencing was performed using the PyroMark Gold Q96 Reagent and the PyroMark ID system (QIAGEN). Pyro Q-CpG™ software v. 1.0.9 was used to measure the optimal order of nucleotide addition, when designing the assays. The software also automatically analyzed the methylation results. Each site is analyzed as a C/T-polymorphism and the percentage of methylation is displayed in a small colored box just above each CpG site, where a 100% denotes a fully methylated C, a 0% denotes an unmethylated C, and intermediate C/T percentages denote partial methylation in the genomic DNA.

### Statistical analysis

Data was analyzed by one-way ANOVA and then posthoc comparisons by using SPSS16 software, and graphs were constructed by using MS-Excel 2010. Results are usually presented as mean ± SD. *P* < 0.05 was considered as significant difference.

## SUPPLEMENTARY FIGURES AND TABLES


