# Supplementary material for: Thymoquinone inhibits cancer metastasis by downregulating TWIST1 expression to reduce epithelial to mesenchymal transition
Source: Oncotarget. 2015 May 19;6(23):19580–91. doi: 10.18632/oncotarget.3973 (PMC4637306; doi:10.18632/oncotarget.3973)
Supplement: Supplementary file 1 [file oncotarget-06-19580-s001.pdf]

SUPPLEMENTARY FIGURES AND TABLES

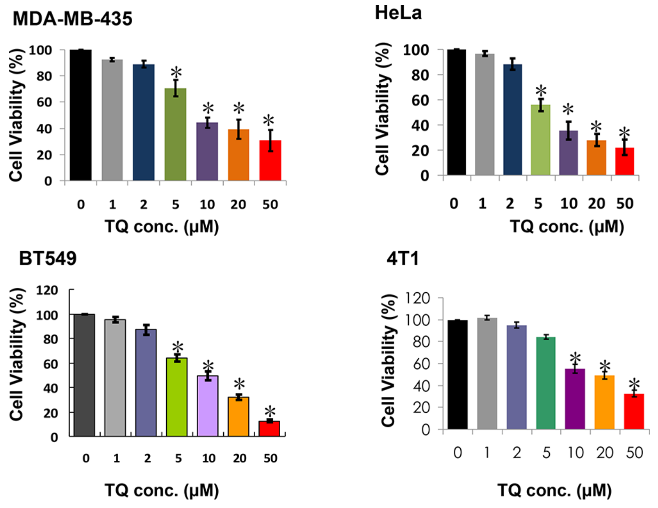

Supplementary Figure S1: Cytotoxic activity of Thymoquinone (TQ) against cancer cells. cancer cells were treated with different conc. of TQ, and cell viability was studied by MTT assay. TQ showed dose dependent inhibitory effect on cancer cell growth. $N = 3$; $^{*}P < 0.05$.

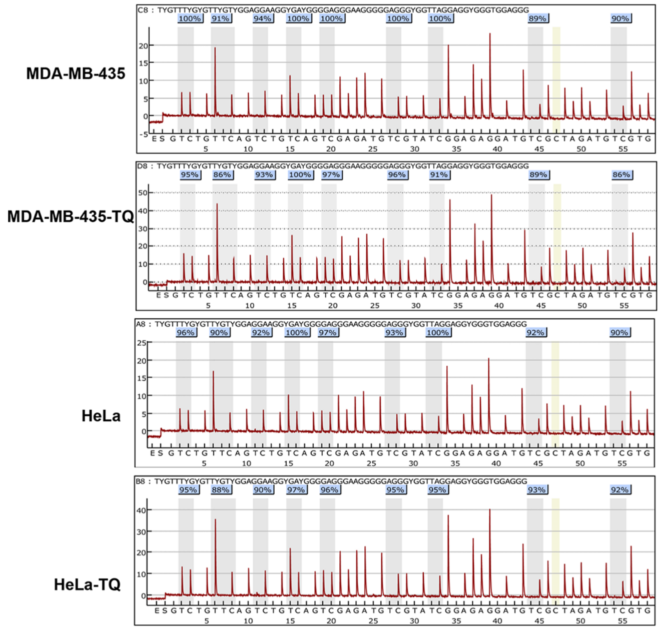

Supplementary Figure S2: Effect of Thymoquinone on *TWIST1* proximal promoter methylation in MDA-MB-435 and HeLa cells. There are no obvious changes in methylation level at *TWIST1* promoter in MDA-MB-435 and HeLa cells without and with TQ treatment. MDA-MB-435-TQ indicates MDA-MB-435 cells with TQ treatment, whereas HeLa-TQ indicates HeLa cells with TQ treatment.

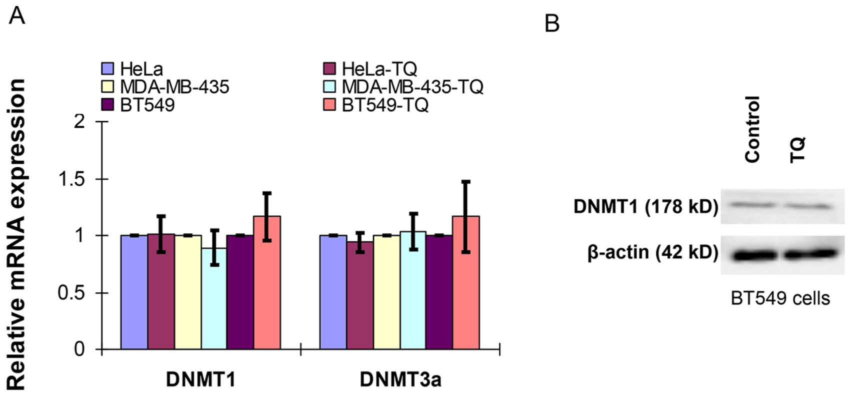

Supplementary Figure S3: Effect of Thymoquinone on expression of DNA methyl transferase 1 and 3a (DNMT1, DNMT3a). DNMT expressions were not changed at mRNA **A.** or protein **B.** level by TQ treatment in cancer cells significantly. $N = 3$; $P > 0.05$.

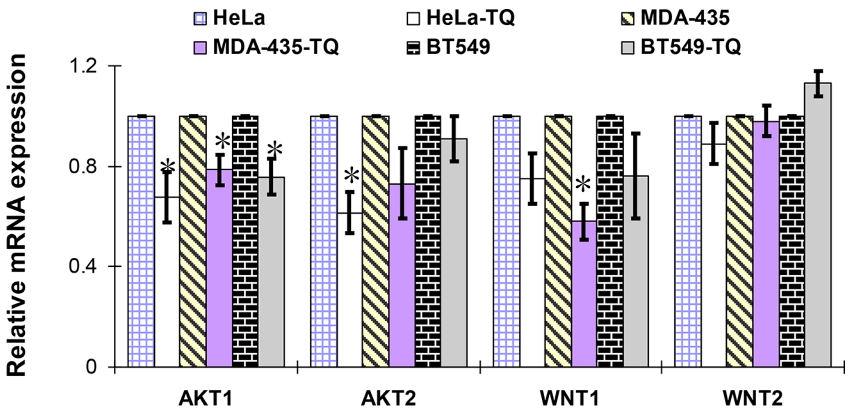

Supplementary Figure S4: Effect of Thymoquinone on Akt and Wnt. Treatment of TQ (5 μM) reduced the mRNA level expression of Akt1 in all of the HeLa, MDA-MB-435 and BT549 cells; Wnt1 in MDA-MB-435 cells; and Akt2 in HeLa cells. $N = 3$; $*P < 0.05$.

Supplementary Table S1: Primer sequences for qPCR used for mRNA isolated from human cancer cell lines

Name	Sequence (5′-3′)
Q18S-48L:	GCAATTATTCCCCATGAACG
Q18S-48R:	GGGACTTAATCAACGCAAGC
Twist1-6L:	GGCATCACTATGGACTTTCTCTATT
Twist1-6R:	GGCCAGTTTGATCCCAGTATT
Snail1-11L:	GCTGCAGGACTCTAATCCAGA
Snail1-11R:	ATCTCCGGAGGTGGGATG
Slug-26L:	TGCACCCTCGGATACCTG
Slug-26R:	ACATTTGGATCACAGAGGCATA
Zeb1-31L:	TGACTATCAAAAGGAAGTCAATGG
Zeb1-31R:	GTGCAGGAGGGACCTCTTTA
E-Cadherin-84L:	TGGAGGAATTCTTGCTTTGC
E-Cadherin-84R:	CGCTCTCCTCCGAAGAAAC
N-Cadherin-68L:	TGGGAAATATAGACAAGCTGGAA
N-Cadherin-68R:	CTGTTATGTTGAGCTCCTCACTGT
Vimentin-56L:	TGGTCTAACGGTTTCCCCTA
Vimentin-56R:	GACCTCGGAGCGAGAGTG

Supplementary Table S2: Primer sequences for qPCR used for mRNA isolated from mouse cancer cell line and tissue

Name	Sequence (5′-3′)
Q18S-48L:	GCAATTATTCCCCATGAACG
Q18S-48R:	GGGACTTAATCAACGCAAGC
Twist1-58L:	AGCTACGCCTTCTCCGTCT
Twist1-58R:	TCCTTCTCTGGAAACAATGACA
Snail1-71L:	CTTGTGTCTGCACGACCTGT
Snail1-71R:	CAGGAGAATGGCTTCTCACC
Slug-71L:	CATTGCCTTGTGTCTGCAAG
Slug-71R:	AGAAAGGCTTTTCCCCAGTG
Zeb1-93L:	AGGTGATCCAGCCAAACG
Zeb1-93R:	GGTGGCGTGGAGTCAGAG
E-Cadherin-79L:	CAGAATGACAACAGGCCAGA
E-Cadherin-79R:	TTCATCACGGAGGTTCCTG
N-Cadherin-18L:	GCCATCATCGCTATCCTTCT
N-Cadherin-18R:	CCGTTTCATCCATACCACAAA
Vimentin-79L:	TGCGCCAGCAGTATGAAA
Vimentin-79R:	GCCTCAGAGAGGTCAGCAAA
